# Preliminary Thermo-Mechanical Evaluation of Fiber Bragg Grating Sensors for Structural Monitoring: Toward Application in Generation IV Nuclear Reactors

**DOI:** 10.3390/mi16111204

**Published:** 2025-10-23

**Authors:** Rocco Contangelo, Carlo Giovanni Ferro, Andrea Bagnasco, Quentin Pouille, Andrea Mazza

**Affiliations:** 1 Department of In-Service Inspection and Repair, *new*cleo, Corso Stati Uniti 38, 10129 Torino, Italy; andrea.bagnasco@newcleo.com (A.B.); quentin.pouille@newcleo.com (Q.P.); andrea.mazza@newcleo.com (A.M.); 2 Department of Mechanical and Aerospace Engineering, Politecnico di Torino, Corso Duca degli Abruzzi 24, 10129 Torino, Italy; carlo.ferro@polito.it

**Keywords:** Fiber Bragg Grating (FBG), femtosecond-FBGs, high-temperature monitoring, stress monitoring, nuclear reactors, Lead-cooled Fast Reactor (LFR), structural health monitoring (SHM)

## Abstract

The performance and longevity of next-generation nuclear reactors depend on the implementation of sensing technologies able to withstand extreme conditions. This study evaluates the performance of femtosecond-laser-inscribed Fiber Bragg Grating (fs-FBG) sensors under simulated startup low-power conditions representative of *new*cleo’s Generation IV Lead-cooled Fast Reactors (LFRs). The primary goal of this preliminary test phase is to validate the suitability of fs-FBG sensors for high-temperature (300 °C) and mechanical stress (16–80 MPa cyclic tensile stress) monitoring, emphasizing their reliability and accuracy. The experimental campaign involved rigorous thermal and thermo-mechanical testing, conducted in compliance with ASTM standards, to assess key performance metrics such as linearity, repeatability, and precision. The results demonstrate that fs-FBG sensors deliver consistent and reliable measurements in extreme environments, with a temperature sensitivity of 12.62 pm/°C and a displacement sensitivity of 3.95 nm/mm. These findings provide a strong basis for the use of fs-FBG sensors in Generation IV nuclear reactors, highlighting their potential as advanced tools for structural health monitoring.

## 1. Introduction

*new*cleo is a pioneering company that is innovating the nuclear industry to play a fundamental role in climate change mitigation and global decarbonization. *new*cleo is developing new Generation IV Lead-cooled Fast Reactors (LFRs), which prioritize safety, efficiency, and sustainability. These reactors leverage the unique properties of lead as a coolant, such as superior thermal conductivity, high boiling point, and effective radiation shielding. The design minimizes the risks associated with water-cooled reactors, such as hydrogen production and core meltdown, while enabling operation at atmospheric pressure, simplifying reactor systems, and enhancing safety. Additionally, *new*cleo’s closed fuel cycle allows the recycling of nuclear waste, reducing environmental impact and optimizing fuel utilization.

Advanced monitoring technologies are essential to ensure the safe and efficient operation of these reactors, as they must withstand extreme operating environments characterized by high temperatures (360–550 °C), mechanical stress, and cumulative radiation from kGy to MGy depending on sensor location. Currently, mechanical and electrical sensors dominate the market due to their broad measurement range, straightforward manufacturing processes, and cost efficiency [1]. Mechanical sensors, such as foil strain gauges, LVDTs, and displacement transducers, are widely used for their maturity, versatility, and ease of implementation. However, in the harsh environments of LFRs, these devices face severe durability challenges. Their rigid designs and limited displacement ranges are prone to thermal drift, hysteresis, and mechanical wear during long-term operation at elevated temperatures [2]. In addition, electromagnetic interference (EMI) originating from reactor control electronics or external fields can corrupt analog signals [3]. To mitigate these risks, comprehensive electromagnetic compatibility (EMC) strategies are required, as outlined by regulatory standards such as RCC-E, which prescribes design rules, qualification testing, and control of electromagnetic disturbances in safety-related instrumentation and control equipment in nuclear power plants. Electrical sensors, particularly thermocouples, are also extensively applied in reactor monitoring, but suffer from similar problems. Prolonged exposure to high temperature and intense neutron/gamma fluxes can induce drift in thermoelectric properties, while insulation materials degrade and cabling becomes increasingly failure-prone under radiation [4]. Like their mechanical counterparts, electrical transducers remain susceptible to EMI/RFI [5], necessitating complex shielding, grounding, and signal conditioning architectures [6]. These limitations highlight the broader drawbacks of conventional transducers in nuclear contexts. Mechanical sensors are hindered by limited displacement ranges and rigid structures, while electrical sensors require intricate circuits and signal processing systems that further compromise reliability. Both categories are inherently vulnerable to external disturbances, including static electricity, EMI, and RFI [1,5]. Collectively, these issues may compromise confidence in the long-term accuracy and robustness of traditional sensor technologies when employed under LFR operational conditions.

To overcome these limitations, fiber optic sensors (FOSs) have gained widespread adoption for detecting and analyzing various physical and chemical parameters [5,7,8]. FOSs provide immunity to EMI, making them ideal for environments with high magnetic fields or radiation [5,8]. Additionally, they are explosion-proof, electrically isolated, and capable of safe operation in cryogenic and hazardous atmospheres. Their lightweight and compact design enables deployment in hard-to-reach locations, while remote sensing capabilities ensure reliable performance over significant distances between sensors and interrogators. Among these technologies, Fiber Bragg Grating (FBG) sensors [9] have emerged as a promising solution due to their small dimensions (external coating diameter smaller than 100 µm [10,11]), lightweight design, and ability to integrate seamlessly with reactor components [12], offering significant advantages over conventional technologies thanks to their minimal volume, flexible geometry, and non-invasive deployment [13]. Unlike traditional sensors, FBGs encode information in the Bragg wavelength, making the measurement indifferent to light power fluctuations. While external references can be used to further improve wavelength accuracy, this is not typically required in standard practice. Their high multiplexing capacity allows multiple sensors—up to 13–14 per fiber, and even more [14,15]—to operate on a single optical line, significantly reducing the number of cables and simplifying installation and network complexity. This feature is especially beneficial for long-distance monitoring or scenarios requiring a large number of sensors. Furthermore, FBGs exhibit remarkable sensitivity across a wide range of measurements, allowing precise detection of minute variations in temperature, strain, and pressure [12]. For instance, they demonstrate a temperature sensitivity of 13 pm/°C and a strain sensitivity of 1.2 pm/µε [12,13].

This study investigates the performance of femtosecond-laser-inscribed Fiber Bragg Grating (fs-FBG) sensors in high-temperature and mechanically stressed environments, representative of reactor startup phase where components experience thermal and mechanical transients at low power in Generation IV LFRs. FOSs are primarily designed to monitor the structural integrity of *new*cleo’s components, such as reactor vessel, control rods, and steam generator. They can provide real-time monitoring of critical parameters like temperature, stress, and structural integrity, enhancing reactor safety and reliability [16,17]. The research focuses on validating the sensors’ linearity, repeatability, and durability under these harsh conditions. To achieve this, a comprehensive testing campaign was conducted, including thermal cycles up to 300 °C and thermo-mechanical tests on metallic specimens with FBGs bonded through high-temperature adhesives. This thermal upper limit is due to machine calibration limitations. However, reaching 300 °C provides a strong basis for our analyses and tests. Future experiments will include additional testing loops at *new*cleo, specifically on molten lead test platforms, to further evaluate sensor performance and reliability.

## 2. Optical Fiber and FBG Sensors

Optical fibers are cylindrical dielectric waveguides designed to transmit light along their axis through the mechanism of total internal reflection. They are composed of three main structural components [18]: the core, typically made of high purity silica and GeO_2_, with the latter added to increase the core refractive index and allow waveguiding; the cladding, also made of silica but with a lower refractive index to confine light within the core and allow its propagation through the principle of total internal reflection; and the coating, usually composed of polymer materials (e.g., polyimide), which provides mechanical protection and resistance to environmental stress. These fibers are widely utilized in sensing technologies due to their immunity to electromagnetic interference, compact size, and multiplexing capability.

Fiber Bragg Gratings (FBGs) represent a great advancement in optical sensing technology. These gratings are inscribed within the core of an optical fiber as a periodic modulation of the refractive index. The periodic modulation in the fiber creates a grating that reflects a specific wavelength, known as the Bragg wavelength ($\lambda_{B}$), while allowing all other wavelengths to pass, thus functioning as a highly selective optical filter. The Bragg’s law is given by

(1) $$\lambda_{B}=2\Lambda n_{\mathrm{eff}},$$

where Λ is the grating period and $n_{\mathrm{eff}}$ is the effective refractive index of the fiber core.

The fundamental mechanism of FBG sensors is based on the principle of wavelength shift, where external perturbations such as strain, temperature, or pressure induce changes in the grating period (Λ) and the effective refractive index ($n_{\mathrm{eff}}$) [9]. These variations result in a shift in the Bragg wavelength ($\lambda_{B}$), expressed as

(2) $$\Delta \lambda_{B}=2\Lambda \Delta n_{\mathrm{eff}}+2n_{\mathrm{eff}}\Delta \Lambda .$$

For practical applications, this relationship can be simplified by introducing coefficients $K_{T}$ and $K_{\varepsilon }$, which represent the temperature and strain sensitivity of the sensor, respectively [19]. The simplified expression becomes

(3) $$\Delta \lambda_{B}=K_{T}\Delta T+K_{\varepsilon }\Delta \varepsilon ,$$

where ΔT is the temperature change, Δε is the strain applied to the fiber, $K_{T}=\lambda_{B}\left(\alpha +\xi \right)$, and $K_{\varepsilon }=\lambda_{B}\left(1-p_{e}\right)$. Here, α is the thermal expansion coefficient, ξ is the thermo-optic coefficient, and $p_{e}$ is the photoelastic constant.

Given that the measured wavelength shift $\Delta \lambda_{B}$ combines contributions from both temperature and strain, effective decoupling strategies are required to accurately extract mechanical strain under varying thermal conditions, such as using multiple FBGs or temperature compensation techniques [9,12]. Dual-FBG configurations, with one sensor bonded to the structure and a second mechanically isolated or wavelength-shifted, allow separation of strain and temperature effects via simple algebraic calculations [20,21]. Interrogator-level compensation exploits opposite slopes of an optical filter to cancel temperature-induced variations [22], while advanced grating designs, including higher-order reflections, tapered sections, or long-period gratings, differentiate strain-induced spectral changes from thermal shifts [21]. Data-driven methods using deep learning models further enable simultaneous extraction of strain and temperature from coupled spectra [23].

### Application of Fiber Optic Sensors

In structural health monitoring (SHM), FOSs enable high-resolution strain, pressure, and temperature measurements. They are used for monitoring underground infrastructure like tunnels [24], assessing landslide stress variations [25], and tracking pore pressure in geohydraulic structures [26]. Additionally, FOSs strengthen structures through fiber-based sensors integrated into carbon materials [27] and enhance leak detection in water infrastructure with active distributed temperature sensing (ADTS) [28]. FBGs can detect pipeline leaks by monitoring temperature and strain [29], while Fabry–Pérot interferometers (FPIs) provide precise pressure monitoring in deep-sea oil wells [30]. Fabry–Pérot sensors are particularly valuable in extreme environments, operating at high temperatures (up to 400 °C) and under intense radiation in nuclear reactors [31]. Their compact design reduces gamma heating, making them suitable for in-pile experiments [32]. These sensors maintain measurement integrity under harsh conditions, crucial for monitoring during events like Loss of Coolant Accidents (LOCA) [33] and for applications in severe nuclear accidents [34].

FOSs are widely used in the nuclear industry, too, for their robustness in extreme environments. Applications include temperature monitoring ensuring safety and process control [35] and detecting pipeline leakages with distributed sensing systems [36]. FBG sensors also demonstrate high radiation tolerance and are under development for next-generation reactors [37,38]. Recent advancements in optical fiber technology have led to the development of radiation-hardened fibers capable of enduring extreme conditions, making them particularly suitable for applications in the nuclear reactors. These fibers are engineered to withstand high temperatures, with femtosecond-laser-inscribed FBGs maintaining reflectivity and spectral stability up to 1000 °C and sapphire-based FBGs showing no degradation up to 1500 °C [39,40]. They are also designed to maintain performance under significant radiation exposure, with research highlighting their resilience against fast neutron fluences up to 10^20^ n/cm^2^s [41] and gamma doses reaching 200 kGy [42,43]. The integration of optical fibers in nuclear reactor designs allows for real-time monitoring of parameters like temperature, strain, and radiation dose rates [44,45], with FBGs proving effective for temperature and strain measurements under challenging conditions [46,47]. Furthermore, the development of sensors capable of detecting acoustic signals and vibrations in fuel assemblies enhances the ability to assess structural integrity and monitor reactor dynamics [48,49]. Studies on the effects of radiation-induced attenuation and luminescence [50,51] reveal significant insights into fiber behavior in high-radiation backgrounds, emphasizing the need for ongoing development to enhance their durability and functionality [52,53,54]. Recent advancements in optical fiber technology not only ensure reliable monitoring in nuclear facilities but also support their potential use in future reactor designs [35,37,55,56].

## 3. Case Study: FBGs for Generation IV Reactors

**Figure 1:**
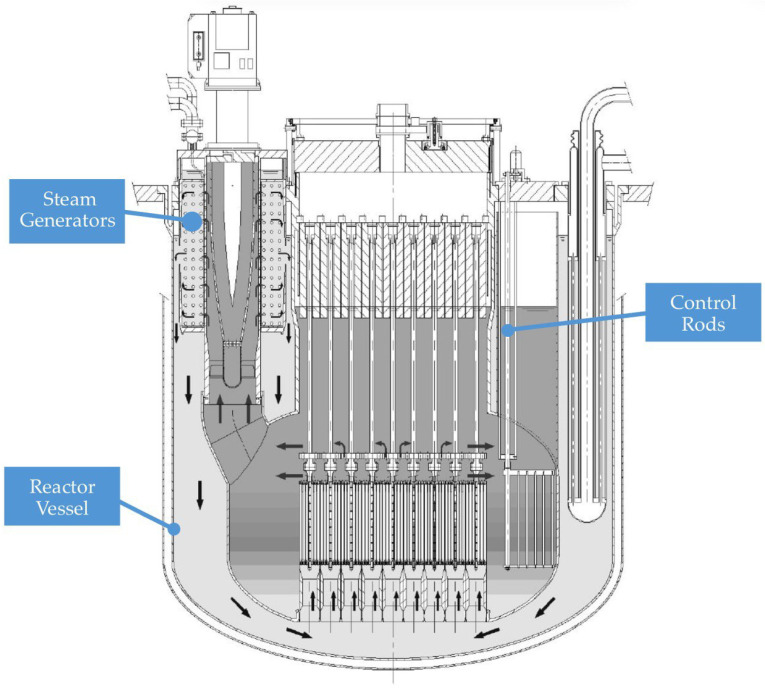
Design schematic of *new*cleo’s LFR-AS-200, highlighting the three key components analyzed in this study: the reactor vessel, control rods, and steam generator.

Part of this study aims to develop an advanced platform for diagnostics and monitoring structural issues in the above mentioned Generation IV nuclear reactors. The platform integrates innovative FOSs, specifically engineered to withstand extreme reactor environments—high temperatures (360–550 °C), radiation exposure (kGy to MGy), neutron flux (approximately 10^12^ to 10^15^ n/cm^2^s), and mechanical constraints. In addition, it incorporates software tools that maximize data extraction and make insights accessible to operators. Since developing innovative and effective sensing technologies for LFRs is a central focus for *new*cleo, FOSs have been identified as one of the most promising solutions for continuous monitoring of critical operational parameters and structural integrity. Their selection is also attributed to their high technology readiness level. FOSs are developed mainly for monitoring parameters for the reactor vessel, control rods, and steam generator structures, highlighted in Figure 1. An in-depth analysis and testing about how and where to place these sensors, which are to be integrated with the actual design of the components, is part of this project.

The reactor vessel (RV) hosts the core and facilitating coolant circulation. The RV consists of a cylindrical shell with a tori-spherical bottom head. It is located inside the reactor building and its flange rests on the support structure all around its circumference. The function is the containment of primary coolant and cover gas. The total height of the vessel is 5140 mm and the wall thickness is 30 mm. Its compact design enhances safety and operability by reducing radioactive waste and eliminating components like shielding and breeding assemblies. Optical fibers with FBG sensors are proposed to be mounted externally on the RV’s wall, avoiding in this case direct exposure to molten lead. Temperature, strain, and vibrations on the RV’s internal surface are inferred using models that correlate external FBG measurements to internal conditions via thermal conduction and elastic wave propagation [49,57]. For durability, optical fibers can be embedded in grooves machined into the RV wall and secured with high-temperature resin. This method (explained and used by Kashaykin et al. [58]) ensures robust sensor installation while shielding the fibers from mechanical and environmental stress. Radiation-hardened fibers with protective ceramic coatings are recommended for nuclear environments [48].

The spiral-tube steam generator (SG) shows *new*cleo’s commitment to compactness, safety, and reliability. It features a thick plate supporting the SG flange, two perforated shells, and a lower plate to support the tube bundle’s weight. The top plate allows vertical tubes to connect to the reactor roof, while the inner perforated shell directs lead flow to the tube bundle and couples with the Amphora-Shaped Inner Vessel (ASIV). The thinner outer shell directs cold lead flow outside the SG. Hot lead enters the SG at approximately 530 °C and, after thermal exchange, exits at around 420 °C, operating at 140 bars. The steam generator converts heat from liquid lead into superheated steam, ensuring efficient heat transfer and preventing lead–water interactions while enhancing safety and structural stability. Optical fibers can be integrated into the SG design through encapsulation into a stainless steel housing and tack-welded [55] onto the tubes, possibly also covered in high-temperature and radiation resistant adhesives [55,59] to ensure perfect adhesion. These FBGs are used to monitor temperature, strain, and vibrations of critical areas of the tubes, for real-time monitoring of their thermo-mechanical behavior and structural health. The tack-welding methodology is explained by Birri et al. [55]. Here, direct contact with molten lead is unavoidable. Alternatively, FOSs can be embedded in grooves machined onto the tube surfaces [57,60], which provides accurate measurements while protecting against molten lead and mechanical damage. High-precision machining or laser technologies are used to create the grooves, with fibers secured via laser welding or encapsulated in durable materials like ceramics or metal alloys. Protective sheaths of stainless steel or ceramics [61] enhance durability, shielding fibers from extreme conditions and structural stresses. While this method improves accuracy and robustness, further research is needed to ensure the mechanical integrity of the tubes and long-term fiber protection [38].

**Figure 2:**
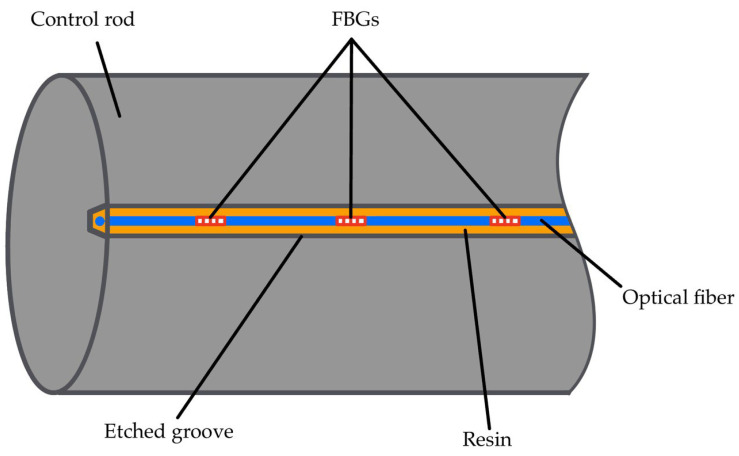
Schematic of a possible optical fiber integration onto a control rod. Optical fiber (blue) is embedded within a machined groove (gray borders), filled with high-temperature, radiation-resistant resin (orange), and possibly encased in a stainless steel tube. The fiber incorporates multiple FBGs (red) for distributed temperature monitoring along the control rod (gray). This multi-layered arrangement ensures protection from molten lead, mechanical stresses, thermal expansion mismatch, and radiation while enabling reliable FBG-based sensing. A similar design, though developed for a different application, was reported by De Pauw et al. [57].

Concluding, control rods (CRs) are used for both reactivity regulation and passive safety. To effectively control the system, six CRs are positioned at the core periphery to manage reactivity changes associated with the transition from cold shutdown to full power, mitigate criticality swings during irradiation cycles due to fuel depletion, and command power variations. These CRs are mechanically actuated through dedicated drive mechanisms, which allows the precise adjustment of their insertion height to control the chain reaction while operating immersed in liquid lead at 530 °C. Optical fibers can be included into the CR design following the same reasoning as that for the steam generator. The most promising solution seems to be embedding them within grooves machined onto their surfaces [57,58], as sketched in Figure 2. The grooves protect the fibers from molten lead and mechanical forces while being filled with high-temperature, radiation-resistant resin to secure and shield the fibers. A stainless steel tube encases the fibers [59], offering enhanced protection against corrosion, thermal shocks, and radiation. A silica micro-capillary tube [62] between the optical fiber and the steel can be further integrated, mitigating thermal expansion mismatches and shielding the fiber from contaminants and stresses. In this configuration, FBGs are used to monitor the temperature distribution along the CRs. This multi-layered setup ensures mechanical robustness, chemical stability, and accurate long-term performance.

### 3.1. FOSs in High Temperature Environments

In moderate temperature ranges (0 °C to 200 °C), FBGs exhibit stable temperature sensitivity, typically 11–13 pm/°C, driven by the thermo-optic effect and fiber thermal expansion [12,13,56]. However, beyond this range, the wavelength–temperature response progressively departs from strict linearity and polynomial calibration becomes necessary for accurate thermometry. More specifically, the observed behavior can be usefully summarized by temperature bands: up to 300–350 °C, the linear model generally remains valid (small nonlinear terms start to appear toward the upper end); in the 300–500 °C window, standard silica FBGs continue to operate but polymeric coatings and adhesives begin to degrade and hydrogen-related instabilities may emerge above 500 °C [63]; between 500 and 700 °C, time-dependent wavelength drift becomes noticeable (an initial red-shift often followed by a slower blue-shift) as draw-induced residual stresses relax and dopant diffusion effects increase [39]; at 700–900 °C, the red-drift phase tends to accelerate with a subsequent rollover toward blue-drift near 900 °C; and at 1000 °C, the red–blue transition is rapid and prolonged grating erasure may occur [39]. These temperature-dependent trends are strongly dependent on waveguide composition and geometry (e.g., higher Ge doping and smaller cladding diameters generally accelerate drift, while larger claddings reduce it), and while pre-annealing improves initial stability, long-term high-temperature service requires careful fiber/coating selection, possible use of fs-inscribed or sapphire gratings for extreme temperatures, and periodic re-zeroing or recalibration [39]. Finally, silica fibers become increasingly embrittled and their protective coatings (e.g., polyimide) degrade more rapidly in the 500–900 °C range, further constraining practical deployment in that band [16]. However, this should not be *new*cleo’s scenario, since temperatures are expected to be in the range of 360–550 °C.

#### Reinforcement Strategies

FBG fabrication techniques, such as the phase-mask method [12] and femtosecond laser inscription [9,12], play a crucial role in determining the grating’s performance. Femtosecond laser inscription in particular enables the creation of highly stable gratings in non-photosensitive fibers, making them ideal for applications in high-temperature and radiation-intense conditions. Fs-FBGs are classified into two main types: Type I and Type II [17]. Type I gratings are formed with laser pulses below the glass damage threshold, inducing densification and a positive refractive index change. In contrast, Type II gratings are produced with laser pulses above the damage threshold, resulting in significant structural changes and enhanced thermal stability, with functionality maintained at temperatures up to 1000 °C [17,39,40]. The use of femtosecond lasers enables the fabrication of gratings stable up to the glass transition temperature of the fiber material, making them ideal for high-temperature applications [16]. In contrast, UV-inscribed FBGs suffer from limitations such as lower thermal stability and the need for photosensitive fibers [12]. Unlike fs-FBGs, which remain stable up to the glass transition temperature, UV-inscribed gratings degrade at significantly lower temperatures, making them less suitable for high-temperature and radiation-intense applications [9,16].

Regeneration techniques have been shown to enhance the thermal stability of fs-FBGs [17,56]. In particular, regenerating ’seed’ FBGs is a promising approach to extending their operational lifetime in high-temperature environments [56,64,65]. This process involves annealing a fully saturated seed grating at high temperatures, up to 950 °C [56,65], leading to the formation of regenerated FBGs (RFBGs) with enhanced thermal stability. Factors such as annealing protocol, fiber composition, and hydrogen sensitization determine the reflectivity and mechanical properties of the regenerated gratings [64,66]. Recent advancements have demonstrated significant improvements, with RFBGs achieving mechanical strengths up to four times greater than standard FBGs and showing operational stability even at temperatures exceeding their regeneration point by over 100 °C [64,65].

RFBGs have proven capable of operating continuously at temperatures above 1000 °C [67], with some demonstrating lifetimes exceeding 9000 h at 890 °C [17,65]. Their dual functionality for temperature and stress–strain monitoring has been confirmed, achieving, respectively, sensitivities of 16.50 pm/°C and 1.25 pm/µε [64]. Despite their robustness, challenges remain, including brittleness induced by the relaxation of internal stresses during regeneration and low reflectivity, which limits their dynamic range [56,64]. Future work should focus on improving annealing methods to mitigate brittleness, developing encapsulation techniques for enhanced mechanical protection, and validating long-term performance under combined thermal, mechanical, and radiative stresses [64,65].

Coatings play a fundamental role in enhancing the performance and durability of FOSs in high-temperature environments [60,61]. Polyimide coatings are widely used for their flexibility and thermal stability but degrade beyond 350 °C, limiting their long-term use in extreme conditions [60]. Metal coatings, such as titanium and zinc, offer superior thermal and mechanical stability above 400 °C, making them more suitable for demanding applications [68]. Titanium-coated FBGs excel in strain sensitivity, while zinc coatings show high sensitivity to temperature, with bimetallic coatings (e.g., Ti/Zn) balancing both properties [68].

For harsher environments, such as nuclear reactors or molten lead-bismuth eutectic (LBE) systems, double-tube protection [59] and ceramic coatings are preferred. Stainless steel tubular housings shield fibers from corrosion and mechanical stress, while ORMOCER coatings resist LBE-induced damage [69]. Additionally, advanced designs using RFBGs with dual-layer protection demonstrate exceptional thermal endurance (up to 1000 °C) and enhanced strain sensitivity, making them ideal for extreme conditions [62].

### 3.2. FOSs in Radiative Environments

Silica-based optical fibers exhibit varying performance degradation due to radiation effects, such as Radiation-Induced Attenuation (RIA), Radiation-Induced Emission (RIE), and Radiation-Induced Refractive Index Change (RIRIC) [44,50,70]. RIA occurs when radiation induces point defects within the silica matrix, increasing light absorption and signal attenuation [50,70]. Fiber composition, particularly doping elements like germanium, phosphorus, and aluminum, influences the severity of RIA, with pure-silica fibers showing superior resistance [50]. Additionally, fibers doped with elements such as germanium exhibit higher sensitivity to radiation-induced defects, while fluorine-doped fibers tend to resist radiation effects better. RIA can be somewhat mitigated through post-irradiation treatments, like hydrogen loading [50]. RIE, on the other hand, results in the generation of light within the fiber during radiation exposure, which, while useful for dosimetry, can interfere with fiber-based communication systems [50]. RIRIC occurs when radiation alters the refractive index of the fiber due to changes in its glass matrix, potentially affecting fiber performance in sensor applications [50,70]. Neutron irradiation contributes to these changes, with neutron flux inducing compaction in the silica matrix, which leads to an increase in the refractive index, affecting sensor accuracy. The signature of displacement damage typically becomes apparent at neutron fluences of 10^16^–10^17^ n/cm^2^s, further influencing the fiber’s optical properties [41,46,71]. In addition, it was observed that radiation-induced Bragg wavelength drift in Ge-doped FBGs tends to saturate under pure gamma irradiation, remaining limited to the equivalent of only a few degrees Celsius, whereas under mixed gamma–neutron fields the drift continues without saturation and can become significantly larger [72].

**Figure 3:**
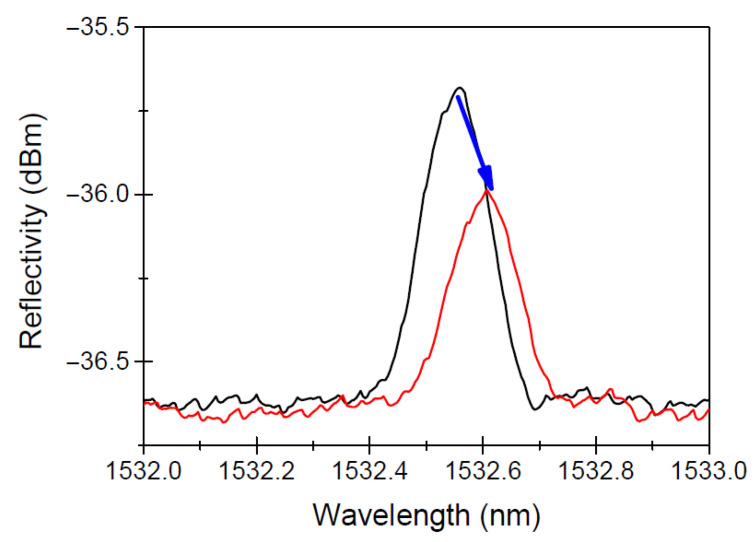
An example of the radiation-induced effects on a Bragg peak reflection spectrum, before (black line) and after (red line) X-ray irradiation at 1.5 MGy at room temperature. The figure highlights the radiation-induced Bragg wavelength shift (RI-BWS) and the reduction of the signal-to-noise ratio, as indicated by the blue arrow. Image reproduced from Morana et al. [70].

When exposed to radiation, FBGs typically suffer from two primary effects [51]: radiation-induced Bragg wavelength shift (RI-BWS) and signal-to-noise ratio reduction [70]; both effects are shown in Figure 3. These effects stem from changes in the optical properties of the fiber due to radiation interactions with the material’s atomic structure. Radiation alters the refractive index of the fiber core, primarily through defect formation in the silica matrix. These defects, sometimes referred to as ’color centers’, absorb light and distort the transmission characteristics of the fiber, leading to wavelength shifts that reduce the accuracy of the sensor. The extent of damage depends on fiber composition and pre- or post-inscription treatments. Radiation-resistant fibers, such as hollow-core photonic bandgap fibers, have demonstrated greater resilience, maintaining low levels of attenuation and retaining sensor accuracy under extreme radiation conditions [73]. Notably, FBGs continue to show resilience even under prolonged radiation exposure, maintaining functionality and temperature sensitivity crucial for nuclear reactor monitoring [47,73]. Experimental investigations confirmed that in low-radiation fields FBGs remain accurate within a few degrees Celsius compared to thermocouples, while at higher neutron fluences and elevated temperatures severe spectral broadening and grating degradation can occur, ultimately limiting sensor performance [72].

The performance of optical fibers degrades when exposed to both high radiation and temperatures [51,52]. RIA increases as both factors accelerate defect formation, with RIA levels varying based on radiation dose and temperature [74]. Understanding this interplay is fundamental for fiber reliability in extreme environments. Temperature’s effect on RIA is dose-dependent. Below 30 kGy, temperature helps anneal defects, but above this threshold, RIA increases with temperature up to 92 °C, after which it plateaus [74]. This suggests that higher temperatures may counteract some radiation damage. Mechanical properties are also affected. Polyimide-coated fibers, which withstand temperatures up to 300 °C, lose mechanical strength when exposed to high radiation doses at elevated temperatures. At 10 MGy (SiO_2_) and 100 °C, their tensile strength drops, limiting their long-term reliability [48,74].

#### Reinforcement Strategies

Reinforcement strategies for optical fibers and FBGs are essential to ensure reliable performance in harsh nuclear environments, where ionizing radiation and elevated temperatures accelerate defect formation, attenuation, and mechanical degradation. A central concern is the RIA, which progressively limits signal transmission and sensor accuracy. To mitigate this effect, different reinforcement approaches have been developed, ranging from optimized fiber core composition to protective coatings and targeted dopant or treatment techniques.

The radiation response of optical fibers is strongly dependent on core composition, dopants, and drawing conditions. Pure Silica Core (PSC) or fluorine-doped fibers are the most radiation-hardened solutions, as they minimize color center formation and maintain RIA levels around 10 dB/km at 1550 nm even after doses of several hundred kGy. These fibers can operate reliably up to MGy dose levels and neutron fluences approaching 10^20^ n/cm^2^s [41]. Radiation-tolerant fibers with germanium-doped cores provide a cost-effective alternative for applications with lower total ionizing doses, such as short data links or lead fibers, where RIA remains limited [75]. Nevertheless, PSC fibers are not universally stable: in some cases they exhibit RIA values up to 2000 dB/km at doses as low as 2 kGy, highlighting the role of fabrication conditions in determining radiation tolerance [41]. Temperature further influences RIA by modifying defect kinetics and recovery, making tailored designs necessary for environments with strong thermal and radiation gradients [75].

Coatings are equally crucial for ensuring both radiation resistance and mechanical integrity. Standard acrylate coatings degrade rapidly under irradiation, while high-temperature acrylates show improved resistance. Polyimide coatings, widely used for environments up to 300 °C, display better radiation tolerance compared to acrylates and aluminum, owing to optimized drawing conditions [74,75]. Polyimide-coated fibers maintain relatively low RIA at moderate doses, but above 10 MGy, particularly at 1625 nm, RIA increases sharply, ultimately limiting long-term signal transmission [74]. Thermal degradation above 350 °C further weakens polyimide coatings, as confirmed by Melin et al. [48]. Aluminum coatings, on the other hand, can withstand higher operating temperatures but increase sensitivity to micro-bending and mechanical fragility.

Rare-earth (RE) doped optical fibers, incorporating elements such as erbium (Er) and ytterbium (Yb), present both challenges and opportunities for radiation hardening [76]. Co-doping with cerium in phosphosilicate cores has proven effective in reducing RIA by mitigating the formation of P1 defects typically associated with phosphorus. Additional reinforcement can be achieved through hydrogen pre-loading, which suppresses defect precursors such as non-bridging oxygen hole centers and converts them into less harmful hydroxyl groups. This significantly lowers RIA, especially in the visible spectrum, and improves fiber stability under gamma irradiation up to cumulative doses of 50 krad [53,76]. Fluorine doping has also been employed as an advanced hardening strategy [53]. While hydrogen treatment offers substantial improvements, it also introduces drawbacks, such as increased radiation-induced luminescence under combined gamma and neutron irradiation, which can interfere with sensing accuracy. Experimental studies show that Bragg wavelength drifts induced by gamma irradiation tend to saturate after a certain dose, suggesting that a deliberate pre-irradiation of sensors can stabilize their long-term response. Conversely, hydrogen-loaded fibers exhibit higher radiation sensitivity and therefore should be avoided in nuclear applications. Standard highly Ge-doped photosensitive fibers without additional treatments demonstrate the lowest radiation-induced drifts [72].

## 4. Materials and Methods

*new*cleo’s operating environment is characterized by high temperatures (360–550 °C) and high cumulated radiation doses (1–10 MGy), combined with a corrosive environment due to molten lead used as coolant. This article presents a comprehensive experimental setup aimed at evaluating, in the first instance, the performance of different FBG sensors under varying thermal and structural conditions. The setup is designed to evaluate the influence of three primary factors—fiber type, wavelength, and gluing technology—on the system’s performance. Similar tests, with lower temperature thresholds, were already conducted in Politecnico di Torino by Aimasso et al. [19,77,78].

The testing setup included four single-mode optical fibers, each 1 m in length, supplied by Télefo S.p.A. The fibers had a core diameter of 9 µm and a cladding diameter of 125 µm (type SM1250SC9/125P), featuring a germanium-doped silica core and a pure silica cladding. The fibers were coated with polyimide. FBGs were inscribed directly into the fiber cores by the supplier using an infrared femtosecond laser (approximately 800 nm wavelength) through the original polyimide coating, preserving mechanical integrity. The laser operated with a pulse duration of approximately 100–300 fs and pulse energy in the range of 200–600 µJ, leading to the formation of Type II gratings, which involve permanent structural changes in the glass matrix due to nonlinear absorption effects. These gratings are known for their high thermal stability and robustness in harsh environments. Each grating was approximately 5–10 mm long, centered in the telecom band (around 1550 nm), with a reflectivity between 54 and 55%, a bandwidth of 0.35 nm, and a side lobe suppression ratio (SLSR) of 30 dB, indicating a clean spectral profile suitable for multiplexing. The fibers’ ID and detailed specifications are listed in Table 1.

The resin utilized to bond optical fibers to the specimens during mechanical tests was WEICON Ceramico W, supplied by WEICON^®^ GmbH & Co. KG (Münster, Germany). All the tests were executed inside the 3119-607 Temperature Controlled Instron chamber of Politecnico di Torino.

**Figure 4:**
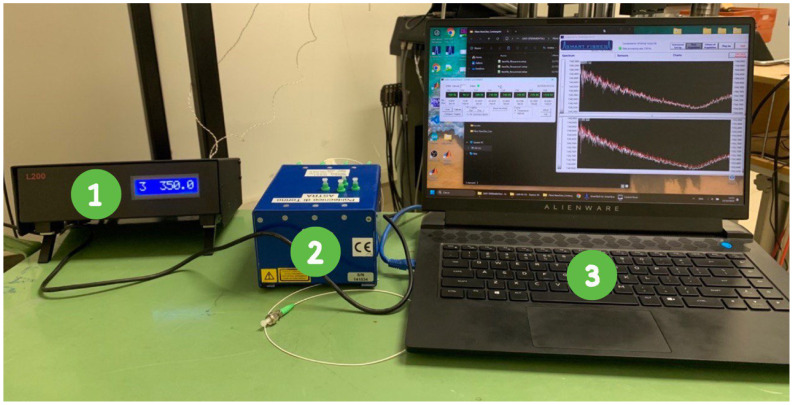
Laboratory setup: (1) thermocouple reader, (2) SmartScan SBI interrogator, (3) control PC.

The interrogation system comprised a SmartScan SBI laser interrogator, capable of monitoring four FBGs simultaneously, transmitting a laser beam, and analyzing the reflected wavelengths. The interrogator was developed by the Smart Fibres company (Bracknell RG12 9BG, UK). This setup minimized data misinterpretations and allowed for independent communication with each sensor. The complete laboratory configuration, illustrated in Figure 4, integrated both FBGs and thermocouples for cross-referencing temperature and strain measurements.

The experimental design involves testing up to four FBGs across multiple wavelengths and gluing methods under controlled thermal cycling and structural loading conditions: the performance of FOSs can vary significantly depending on the type of fiber, wavelength of operation, and the gluing technology used for sensor integration. The goal is to identify the most promising fiber optic sensors for industrial applications that require high temperature and structural resistance.

Each test is conducted with two repetitions: the first one establishes a baseline for consistency, while the second serves to confirm the reliability of the results. This approach is sufficient for thermal cycles because the nature of thermal processes tends to produce consistent outcomes with minimal variation, as experienced by Madan et al. [60,61]. The relatively stable behavior of materials during thermal cycling means that two repetitions can effectively capture any significant trends or deviations without the need for additional trials. Future research and testing will be conducted, with the main focus on testing fibers in radiative environments [58,63], to evaluate the responsiveness and degradation of the sensors under gamma radiation [16,42,45] and neutron flux [47,71].

### 4.1. Thermal Testing of Free Fibers

**Figure 5:**
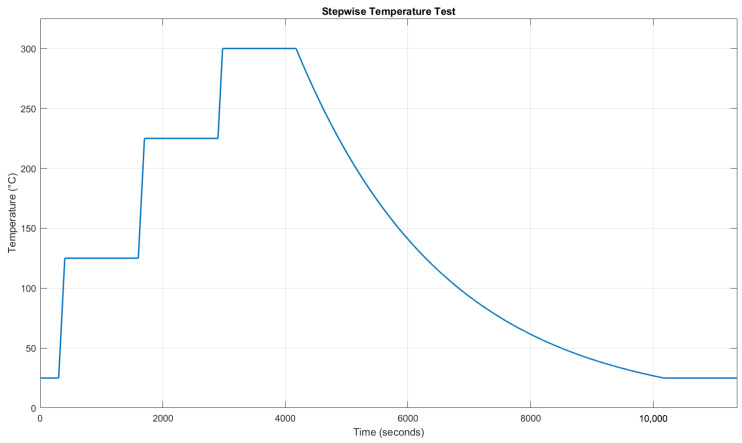
Stepwise temperature cycle example. Here, proposed a thermal test with a heating rate of 60 °C/min is proposed, including a holding phase of 20 min every 100 °C. Once the maximum operating temperature is reached, the chamber is turned off to allow it to naturally cool down to room temperature (this is an estimate close to the required time for cooling).

Two thermal tests were conducted inside the Instron thermal chamber, capable of reaching temperatures up to 300 °C. Fibers were placed on a neutral platform and subjected to step-wise thermal cycles with heating rates of 60 °C/min and holding phases of 20 min at every 100 °C. The ideal thermal cycle to be executed is shown in Figure 5.

The goal is to evaluate the linearity and stability of the FBG wavelength response under repeated thermal variations. Our fs-FBGs are expected to have a better linearity along a wider range of temperature [60,61]: the grating structures inscribed using femtosecond lasers have attractive annealing characteristics and may provide a long-term stable operation in high-temperature environment compared to conventional UV laser written grating structures. K-type and T-type thermocouples are employed as temperature reference sensors. K-type thermocouple has a sensitivity of 41 µV/°C and an accuracy of ±2.5 °C, while the T-type presents a sensitivity of 43 µV/°C and an accuracy of ±1.0 °C. The thermocouples reader samples data at 1 Hz, with a resolution of 0.01 °C. The FBG interrogator acquires data at 2.5 Hz for enhanced precision.

### 4.2. Thermal-Structural Testing

The second testing phase focused on evaluating the combined effects of thermal and mechanical loads on the performance of fs-FBGs. Optical fibers were bonded to tensile test specimens through resin and were subjected to cyclic mechanical stress both at room temperature and higher temperatures. The elongation data of the specimens were recorded to analyze deformation patterns. These data were acquired through the Instron machine that recorded the vertical movements of its crosshead. Thermal cycles similar to those utilized in the previous phase were employed, allowing for a controlled analysis of sensor response under simultaneous thermal and mechanical stresses.

Conducted within the Instron thermal chamber, this phase utilizes a maximum temperature of 250 °C (due to resin’s limitation) and controlled load cycling ranging from 1 kN to 5 kN, corresponding to nominal stresses in the dogbone specimen of $\sigma_{n}=F/A\approx 16–80\;\mathrm{MPa}$, where $A=62.5\;{\mathrm{mm}}^{2}$ is the cross-sectional area of the reduced gauge section. As these stress levels are well below the yield strength of the material, the specimen response is fully within the linear elastic regime, and the corresponding strain ranges can be deduced as $\varepsilon =\sigma_{n}/E$. Under this condition, the hysteresis introduced by cyclic loading is negligible, because the material remains well within the elastic domain. At room temperature, using E=193GPa for AISI 316 L(N), the applied stress range translates into a strain interval of approximately $\varepsilon_{25\;{}^{\circ}C}=83–415\;µ\varepsilon$. At elevated temperature (250 °C), where the elastic modulus decreases to E(250)=167GPa according to $E\left(T\right)=-\frac{4}{35}T+195.7$ [GPa] (elastic modulus linear relationship taken from https://www.acciaiterni.it/mercato-e-prodotti/acciaio-inossidabile/proprieta/, accessed on 29 August 2025), the same stress range corresponds to a slightly higher strain interval of $\varepsilon_{250\;{}^{\circ}C}=96–479\;µ\varepsilon$.

Since the specimen response is fully within the linear elastic regime, and assuming uniform strain within the gauge section (neglecting machine compliance), the computed strain ranges can be translated into specimen elongations along the gauge length $L_{\mathrm{gauge}}=17.50\;\mathrm{mm}$. This yields elongation intervals of $\Delta L_{25\;{}^{\circ}C}=0.0014–0.0073\;\mathrm{mm}$ at room temperature and $\Delta L_{250\;{}^{\circ}C}=0.0017–0.0084\;\mathrm{mm}$ at 250 °C. Here, the lower and upper bounds correspond to the minimum and maximum applied stresses (16 MPa and 80 MPa), defining the full cyclic displacement range experienced by the specimen. From a technical point of view, the total elongation span $\Delta L_{\mathrm{range}}=\Delta L_{\max}-\Delta L_{\min}$ quantifies the full displacement excursion, providing a direct link between applied stresses and the resulting deformations for the sensor-monitored section. This range is $\Delta L_{\mathrm{range},\;25\;{}^{\circ}C}=0.0059\;\mathrm{mm}$ and $\Delta L_{\mathrm{range},\;250\;{}^{\circ}C}=0.0067\;\mathrm{mm}$, which represents the maximum cyclic elongation to which the FBGs are subjected during the tests.

This specific type of mechanical test was chosen to assess the FBG sensors’ ability to withstand and accurately measure mechanical stresses. During reactor operation, components are subjected to dynamic stresses and vibrations due to thermal expansion, mechanical loading, and fluid-induced forces. The present cyclic protocol does not replicate the exact stress amplitudes, strain rates, or load spectra expected in molten lead environments; rather, it serves as a proxy to demonstrate the sensors’ mechanical robustness and stability under repeated deformation. The test thus represents a preliminary step, complementing previous studies on vibration effects in lead-cooled systems [49,79], while more representative load conditions will be addressed in subsequent dedicated campaigns.

Two main tests were conducted:

- Tests at room temperature: Three sessions of 3000 cycles at 5 Hz, conducted without heating.
- Tests at elevated temperatures: Six phases, alternating between temperature ramping and stabilization. First, the temperature is gradually increased (mechanical loads consisting of 800 cycles at 1 Hz, with a heating ramp rate of 20 °C/min). Once the target temperature is reached, a stabilization phase follows, maintaining the temperature for 3000 cycles at 5 Hz. This sequence is repeated three times, reaching 100 °C, 200 °C, and finally 250 °C, resulting in a total of three ramp phases and three stabilization phases.

While only two main tests were conducted to limit the number of sensors and specimens, additional repetitions could enhance data reliability. Resin adhesion at high temperatures was crucial to ensure sensor stability. This testing phase offers valuable insights into the long-term performance of FBG sensors under realistic thermo-mechanical loading conditions.

**Figure 6:**
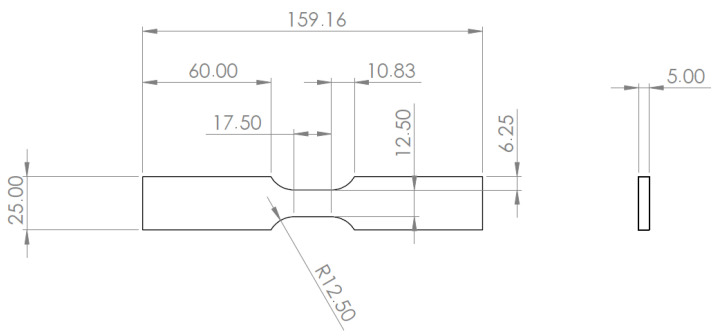
Dog-bone tensile specimen draft, designed following ASTM E606 dimensions.

The test specimens, illustrated in Figure 6 and designed following the ASTM E606/E606M-21 Standard [80], were fabricated from AISI 316L(N) stainless steel, matching materials used in *new*cleo’s Generation IV reactor components. Optical fibers were bonded on the plane surface of the tensile test specimens using the selected high-temperature resin.

**Figure 7:**
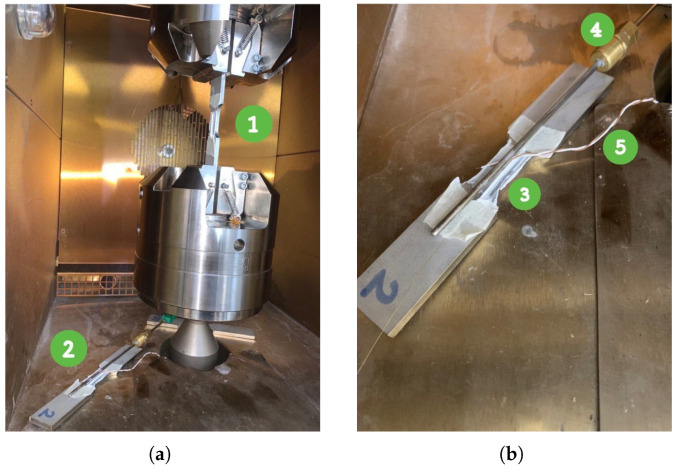
Experimental setup for the thermo-mechanical tests: (**a**) General view inside the Instron chamber: (1) Specimen 1, subjected to combined tensile loading and thermal cycling, equipped with FBG4 for the first test and with FBG1 for the second; (2) Specimen 2, used as a reference to monitor temperature-only response and equipped with FBG2 throughout all tests. (**b**) Zoomed view of Specimen 2: (3) FBG2, (4) the K-type thermocouple, and (5) the T-type thermocouple.

The setup for this testing phase is illustrated in Figure 7. It includes two primary specimens:

- Specimen 1 (labeled as (1) in Figure 7a) is equipped with optical fibers and undergoes both thermal and mechanical cycling. FBG4 was mounted for the first test, followed by FBG1 for the following second test. This specimen is used to study the combined effects of temperature and mechanical stress on the fibers’ performance.
- Specimen 2 (labeled as (2) in Figure 7a) has FBG2 attached with resin and is dedicated to thermal measurements only. This specimen serves as a thermal reference for both the first and second tests, remaining isolated from mechanical stress.

To enhance the accuracy of temperature readings, Specimen 2 is also equipped with K-type and T-type thermocouples (labeled as (4) and (5) in Figure 7b), which provide precise temperature reference values for the measurements from FBG2 (labeled as (3) in Figure 7b). The displacement data acquired from the FBG were compared to that registered from the Instron machine that registered its crosshead vertical movements with a sensitivity of 10 µm/kN, along with a resolution of 1 µm and accuracy of ±0.03% of the reading.

### 4.3. Resin’s Selection and Application

The choice of resin is critical for ensuring the reliable integration of optical fibers with the test specimens. Resins provide a robust bond capable of withstanding high-temperature and mechanical stresses while preserving the optical performance of the sensors. In this study, WEICON Ceramico W was selected due to its superior thermal resistance (up to 250 °C), high tensile strength (54 MPa), good Young’s modulus (9.4–10 GPa), and excellent adhesion properties. The mixture consists of an epoxy base with aluminum oxide as the filler material, mixed in a ratio of 100:25 by weight.

**Figure 8:**
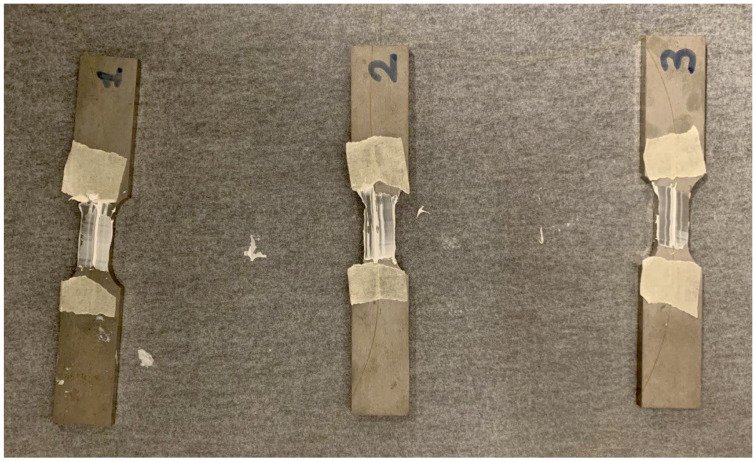
Resin (WEICON Ceramico W) application onto the tensile specimens, with the optical fibers fixed in position using paper tape. A fine applicator was used to deposit a uniform layer (0.1–0.2 mm) along the fiber, minimizing stress concentrations and avoiding damage. The resin was cured for 24 h before testing. The FBGs are positioned at the center of the resin-coated area for accurate wavelength measurements. From left to right, the bonded FBGs are FBG4, FBG2, and FBG1.

The resin deposition process was carried out manually using the fine applicator supplied with the resin, which is ergonomically designed to allow a precise and damage-free application along optical fibers. Prior to resin application, each fiber was temporarily secured to the specimen using paper tape to maintain its position and alignment during the process. The resin was then carefully applied along the length of the fiber, taking care to avoid air bubbles, excessive accumulation, or mechanical damage to the fiber. A very thin layer of resin, approximately 0.1–0.2 mm thick, was deposited directly on the optical fiber to bond it to the specimen. A small amount of resin spread onto the sides, covering part of the gauge section of the tensile specimen, but the effective bonding layer on the fiber remained within this nominal range. The resin was then left to cure for 24 h under ambient conditions, resulting in stable adhesion of the fiber to the specimen. This method ensured a uniform layer of resin, minimizing localized stress concentrations that could distort the optical signal and introduce errors in wavelength measurements [81], as illustrated in Figure 8. The FBGs bonded to the tensile test specimens in the figure are identified, from left to right, as FBG4, FBG2, and FBG1, and the uniform application contributed to accurate and reliable measurements under the thermal and mechanical loads applied in the tests.

## 5. Results and Discussion

In the following sections, the results from the thermal test are discussed, starting with the linear calibration of the FBG sensors, followed by an analysis of temperature estimation using these sensors. In the thermal-structural test section, the correlation between wavelength and displacement is explored, along with a compensation technique for temperature effects. Finally, the displacement estimation through FBG sensors is presented, highlighting how these sensors perform under combined thermal and mechanical stress.

These tests are important because they show how dependable and versatile FBG sensors can be. The results suggest that, with proper calibration, FBG sensors can effectively measure both temperature and strain, making them suitable for high-stress, high-temperature situations. The positive findings also open up possibilities for future studies, which could involve more complex conditions, such as working in a radiative environment.

### 5.1. Thermal Test Results

**Figure 9:**
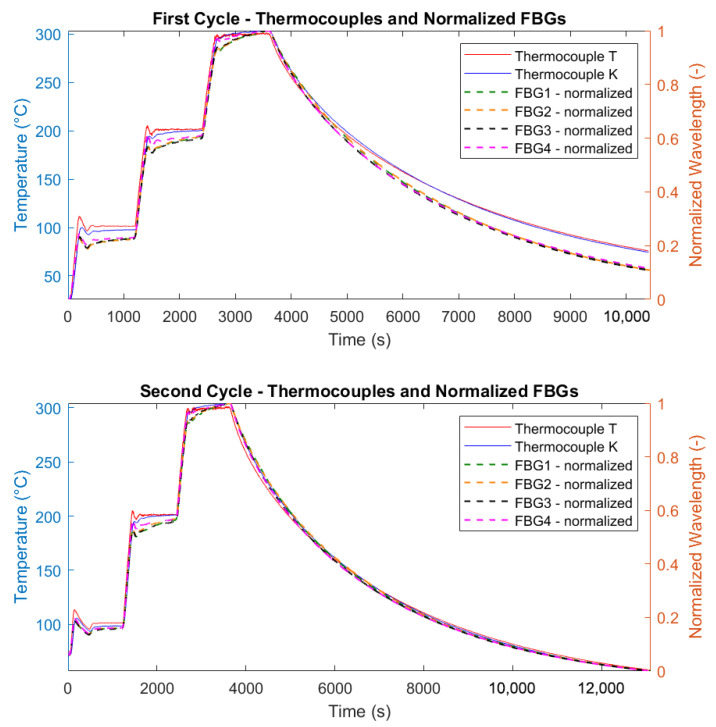
Normalized correlation between temperature changes (solid lines, left blue axis) and wavelength shifts (dashed lines, right orange axis) for the two thermal cycles: the upper plot corresponds to the first cycle and the lower plot to the second cycle. The left vertical axis reports the temperature evolution, and the right vertical axis reports the normalized wavelength displacement.

Two stepwise thermal tests were performed, according to Figure 9, to understand the correlation between the Bragg wavelength shifts and temperature variations. The temperature-induced shift in the Bragg wavelength ($\lambda_{B}$) is described by Equation (1), where both $n_{\mathrm{eff}}$ and Λ are affected by temperature through thermal expansion and the thermo-optic effect. The simplified relationship between the wavelength shift ($\Delta \lambda_{B}$) and temperature variations (ΔT) is expressed as

(4) $$\Delta \lambda_{B}=K_{T}\Delta T,$$

where $K_{T}$ is the temperature sensitivity coefficient determined through calibration. To standardize the analysis, the wavelength data from the FBGs were normalized using min–max scaling:

(5) $${\tilde{\lambda }}_{B}=\frac{\lambda_{B}-\lambda_{\min}}{\lambda_{\max}-\lambda_{\min}},$$

where $\lambda_{B}$ is the instantaneous Bragg wavelength, and $\lambda_{\min}$, $\lambda_{\max}$ are the minimum and maximum values within each dataset. This normalization yields dimensionless values between 0 and 1 for a direct comparison across the four FBGs. The results of Figure 9 demonstrate a consistent linear correlation between temperature changes and wavelength shifts.

It is important to highlight the influence of the chamber’s fan on the FBG measurements. Specifically, during the cooling phase of the first test shown in Figure 9, when the fan was active, a small drift in the FBG wavelengths occurred relative to the temperature readings. In contrast, during the second test, when the fan was turned off, the alignment between the FBG wavelengths and temperature measurements was nearly perfect.

**Figure 10:**
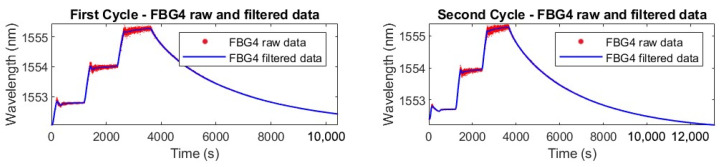
Filtered response of FBG4 during thermal cycling. The left figure corresponds to the first heating–cooling cycle, while the right one corresponds to the second cycle. In both cases, the plots show the raw FBG data acquired from the interrogator (red) and the corresponding signal after applying a Moving Average Filter (blue), for noise reduction and improved signal clarity.

For the experiments, raw wavelength data from the FBGs were processed using a Moving Average Filter (MAF) to reduce noise and enhance data clarity. The MAF was implemented in MATLAB (version 2023a), with a window size of 150 data points used to calculate the smoothed values. As a side effect, this smoothing introduced a small time delay in the FBG response, which accounts for the apparent lag in Figure 9 relative to the thermocouple traces during isothermal conditions. In particular, the Moving Average Filter used N=150 samples at an interrogator sampling rate of 2.5 Hz (Δt=0.4 s) introducing a causal group delay of (N−1)/2·Δt≈149/2·0.4≃29.8 s, fully explaining the observed lag. Figure 10 illustrates the comparison between raw and filtered data for two thermal cycles of FBG4.

#### 5.1.1. Linear Calibration

**Figure 11:**
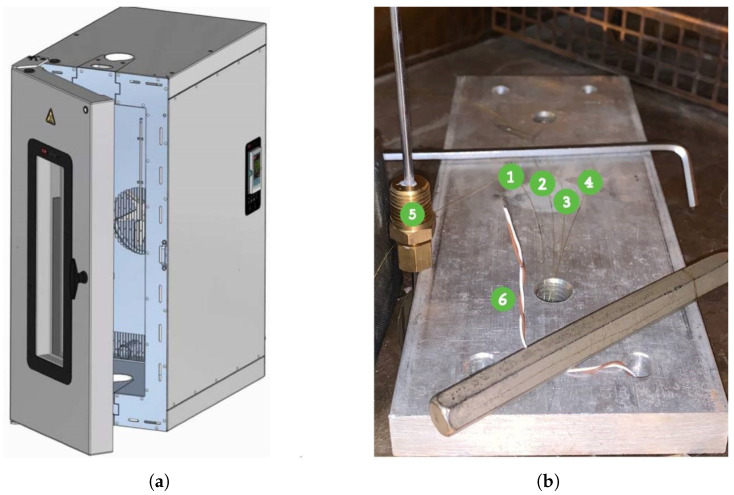
(**a**) 3119-607 Temperature-Controlled Instron Chamber. (**b**) Setup of the optical fibers displaced on a neutral platform, to be inserted inside the Instron chamber: (1) FBG1, (2) FBG2, (3) FBG3, (4) FBG4, (5) K-type thermocouple, (6) T-type thermocouple.

The scope of this linear calibration is to establish a precise and reliable relationship between temperature and Bragg wavelength shifts, enabling accurate temperature measurements in high-temperature environments. This approach is based on the assumption that the relationship between the Bragg wavelength shift ($\Delta \lambda_{B}$) and the corresponding temperature change (ΔT) is linear and can be accurately described by Equation (4). Therefore, the aim of the linear calibration is also to determine the temperature sensitivity coefficient $K_{T}$. To validate this assumption and determine $K_{T}$, a controlled thermal environment was established where the FBG sensors and a T-type thermocouple were positioned in close proximity to ensure uniform thermal exposure and minimize spatial temperature gradients. Temperature was increased following the described stepwise thermal cycle (heating phases followed by stabilization phases) up to 300 °C, with the thermocouple providing precise reference measurements while the FBG sensors concurrently recorded the corresponding Bragg wavelength shifts, $\Delta \lambda_{B}$. To ensure high measurement accuracy, the T-type thermocouple was selected due to its superior stability and proximity to the FBG sensors during testing, effectively minimizing discrepancies arising from spatial temperature gradients. As depicted in Figure 11, the tip of the T-type thermocouple was positioned only a few millimeters away from the FBGs, whereas the K-type thermocouple was located several centimeters away, leading to potential deviations in recorded temperature values.

**Figure 12:**
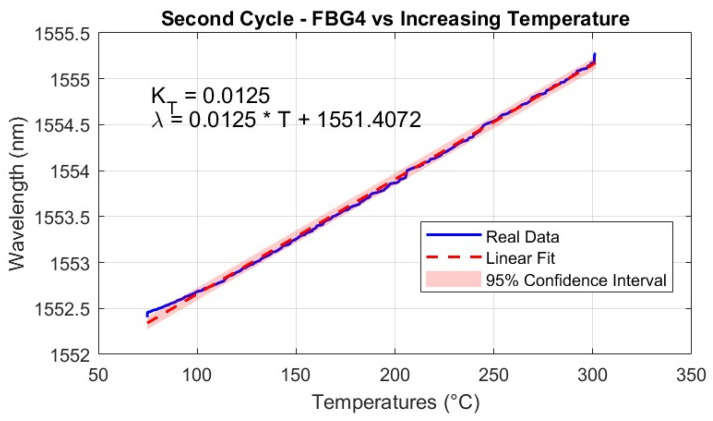
Linear regression of the wavelength shift of FBG4 as a function of temperature during the second heating cycle. The red dashed line represents the linear fit, while the blue line shows the measured wavelength shift data, with the shaded region indicating the 95% confidence interval. Small deviations in the data around 100, 200, and 300 °C correspond to stabilization steps, where the temperature was held constant to ensure thermal equilibrium before continuing the heating ramp.

The calibration procedure consisted of plotting the measured wavelength shifts against the temperature variations and performing a linear regression analysis, where the slope of the best-fit line directly provided the value of $K_{T}$. The validity of the linear model was confirmed by a high coefficient of determination ($R^{2}\approx 1$). The precision of the fit was further substantiated by the narrow 95% confidence intervals, as shown in Figure 12, which underscore the reliability of the linear fit, even when extrapolated to higher temperature ranges. The small jumps that appear in Figure 12 at 100, 200, and 300 °C are due to the thermal stabilization steps performed during the heating cycles. During these steps, the temperature was held constant for 20 min to allow the system to reach thermal equilibrium, which caused small deviations in the measured sensor responses, as the materials adjusted to the stable temperature conditions. The temperature sensitivity coefficients $K_{T}$ were determined using temperature readings from the reference thermocouple. Therefore, the wavelength shift of each FBG was correlated with the temperature measured by the T-type thermocouple. This approach eliminates potential biases from the interrogator’s internal calibration, providing a direct sensitivity assessment based on the actual thermal conditions experienced by the sensors. However, this assessment does not account for potential measurement and calibration uncertainties associated with the T-type thermocouple, as these values are not known.

The temperature sensitivities of each FBG, for both first and second cycles, are highlighted in Table 2. These sensitivity values are in line with studies and tests for temperature monitoring through FBGs [14,56,60,61,62,64].

Additionally, the use of fs-FBGs, which are known for their enhanced thermal durability, ensured a stable and predictable relationship between temperature and wavelength shifts across the operational range. It is important to note that while the linear calibration remains valid up to approximately 350–400 °C, beyond this range, the sensor response may exhibit nonlinearity, necessitating the adoption of polynomial regression models to accurately characterize the temperature dependence.

#### 5.1.2. Temperature Estimation Through FBG Sensors

Using the calibrated temperature sensitivity coefficient ($K_{T}$), FBG sensors can predict temperature values based on the measured wavelength shifts. The predicted temperature is

(6) $$\Delta T_{\mathrm{predicted}}=\frac{\Delta \lambda_{B}}{K_{T}}.$$

**Figure 13:**
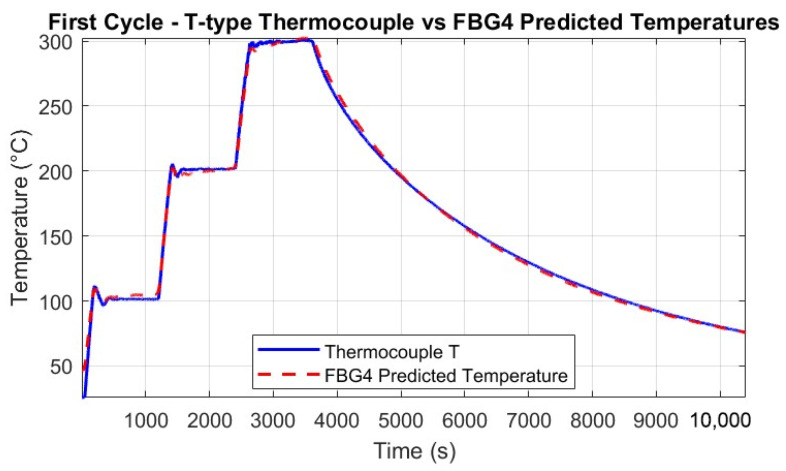
Predicted temperatures from FBG4 (red dashed line) compared to T-type thermocouple measurements (blue solid line) during the first thermal cycle. The figure illustrates the accuracy of the FBG calibration process in capturing temperature-induced wavelength shifts, with minor deviations due to fiber placement and small vibrations in the thermal chamber.

**Figure 14:**
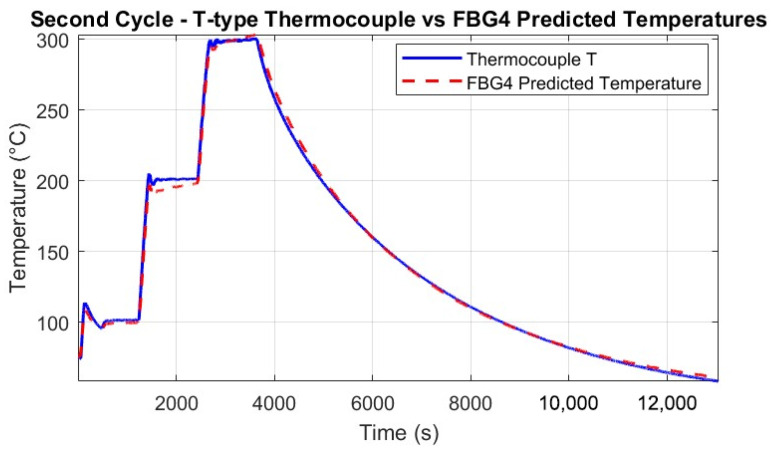
Predicted temperatures from FBG4 (red dashed line) compared to T-type thermocouple measurements (blue solid line) during the second thermal cycle. The figure confirms the repeatability of the FBG response and the reliability of the calibration procedure, with small discrepancies caused by slight differences in sensor positioning and thermal chamber disturbances.

Throughout the thermal tests, the predicted temperatures were computed for all FBGs and compared against the reference T-type thermocouple measurements. Figure 13 and Figure 14 show the good agreement between predicted and measured temperatures for the single FBG4 in both thermal cycles, highlighting the accuracy of the calibration process.

The alignment with thermocouple measurements confirms the calibration process and the FBG’s precision in detecting temperature-induced wavelength shifts. Minor discrepancies in some sections may be attributed to the setup, where the thermocouples and FBGs were not perfectly co-located, and to small vibrations of the fibers caused by the thermal chamber’s fan.

### 5.2. Thermal-Structural Test Results

The thermal-structural test evaluated the performance of fs-FBG sensors in measuring strain and displacement under combined thermal and mechanical loads, simulating nuclear reactor conditions. Specimens, made of AISI 316L(N) stainless steel, were subjected to cyclic mechanical stress at room and elevated temperatures (250–300 °C), corresponding to nominal stresses of 16–80 MPa in the gauge section. The tests, performed in the Instron chamber, assessed the sensors’ ability to withstand dynamic stresses, vibrations, and thermal expansion. Resin adhesion was crucial for sensor stability, and displacement data of the specimen were recorded to analyze deformation.

Two main test phases, as described in Section 4.2, were conducted: room temperature cycling (3000 cycles at 5 Hz) and elevated temperature cycling (alternating between gradual heating, 800 cycles at 1 Hz, and constant temperature, 3000 cycles at 5 Hz). Specimen 1, equipped with an optical fiber, was subjected to both thermal and mechanical cycling, while Specimen 2, used for thermal measurements, served as a reference with K-type and T-type thermocouples. In the first test phase, FBG4 was used for the mechanical tests and was bonded to Specimen 1. In the second test phase, FBG1 replaced FBG4 for the mechanical tests on Specimen 1. Throughout both test phases, FBG2 served as the thermal reference sensor and was bonded to Specimen 2.

#### 5.2.1. Correlation Between Wavelength and Displacement

The relationship between the FBG wavelength shift ($\Delta \lambda_{B}$) and displacement is governed by strain (Δϵ) and temperature (ΔT). For purely mechanical effects, the wavelength shift is expressed as

(7) $$\Delta \lambda_{B}=K_{\epsilon }\Delta \epsilon ,$$

where $K_{\epsilon }$ is the strain sensitivity coefficient. Under combined thermal and mechanical conditions, the total shift includes both strain and temperature contributions:

(8) $$\Delta \lambda_{B}=K_{T}\Delta T+K_{\epsilon }\Delta \epsilon ,$$

where $K_{T}$ is the temperature sensitivity coefficient. The terms $K_{T}\Delta T$ and $K_{\epsilon }\Delta \epsilon$ represent the respective contributions of temperature and strain, both of which must be considered to ensure accurate displacement measurements.

**Figure 15:**
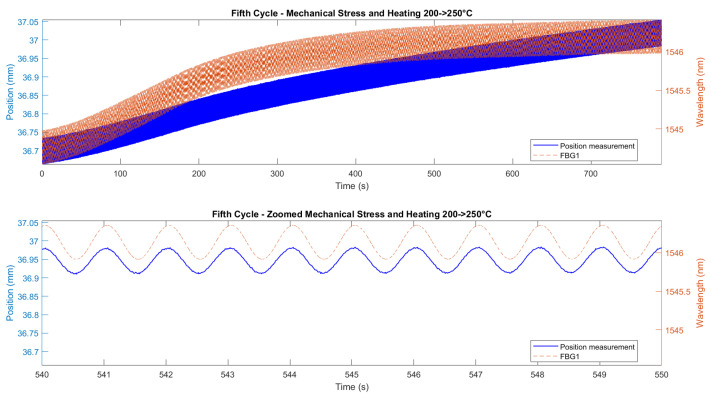
Correlation between FBG1 wavelength shift and Instron crosshead displacement during the fifth cycle of the second test (temperature ramp 200–250 °C, cyclic loading 1 Hz). Top panel shows the full cycle; bottom panel shows a zoomed interval. Blue solid line (left blue axis): Instron crosshead displacement. Orange dashed line (right orange axis): FBG wavelength shift (contains both temperature and stress contributions). A near-constant 20–30 ms time offset between the signals is observed, likely due to the viscoelastic response of the embedding resin and small synchronization differences between acquisition systems.

**Figure 16:**
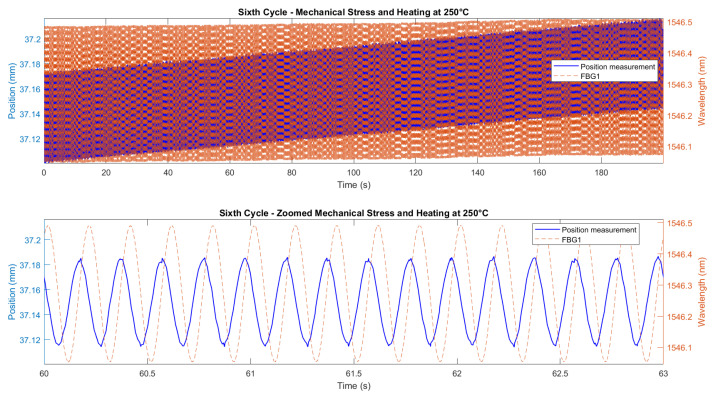
Correlation between FBG1 wavelength shift and Instron crosshead displacement during the sixth cycle of the second test (constant 250 °C, cyclic loading 5 Hz). Top panel shows the full cycle; bottom panel provides a zoomed interval for clarity. Blue solid line (left axis): Instron crosshead displacement. Orange dashed line (right axis): FBG wavelength shift (contains both temperature and stress contributions). A 20–30 ms offset, attributed to resin viscoelasticity and minor timing differences, is visible here, too; dynamic trends and amplitudes remain consistent.

Figure 15 and Figure 16 demonstrate the correlation between the wavelength shifts of FBG1 and the position measured by the Instron chamber. In the fifth cycle (Figure 15), the temperature increased from 200 °C to 250 °C under cyclic loading at 1 Hz, while the sixth cycle (Figure 16) maintained a constant temperature of 250 °C with cyclic loading at 5 Hz. A static shift of 20–30 ms was observed between the FBG and Instron signals. This offset is likely due to a combination of the viscoelastic response of the embedding resin, which slightly delays strain transfer, and minor synchronization discrepancies between the FBG interrogator and the Instron acquisition system. While the dynamic trends and peak-to-peak values are consistent, this delay results in a constant offset between the curves.

#### 5.2.2. Temperature-Effect Compensation

**Figure 17:**
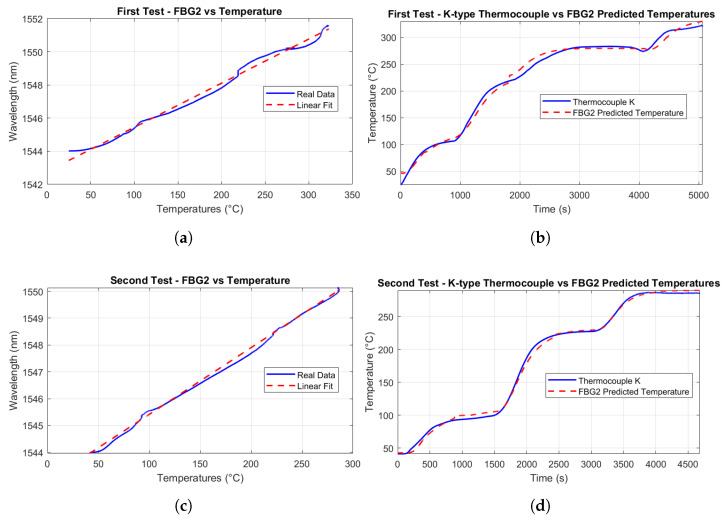
Thermal calibration of FBG2. (**a**) Correlation between temperature and Bragg wavelength shift during the first calibration test, with experimental data (blue line) and linear regression fit (red dashed line). (**b**) Temperature values predicted from FBG2 during the first test (red dashed line) compared to reference measurements from a K-type thermocouple (blue line). (**c**) Correlation between temperature and Bragg wavelength shift during the second calibration test, again showing raw data (blue line) and linear fit (red dashed line). (**d**) Predicted temperatures obtained from FBG2 during the second test (red dashed line) and comparison to the K-type thermocouple measurements (blue line).

To isolate strain-induced wavelength shifts from temperature effects, a second FBG (FBG2) was used as a reference sensor, sensitive only to temperature changes. Positioned on a non-loaded specimen, FBG2 was calibrated using the same procedure followed during the thermal test, with the results shown in Figure 17. Among all available fibers, FBG2 was selected because its thermal response most closely matches those of FBG1 and FBG4 when calibrated against each other. FBG1 and FBG4 exhibited temperature-induced wavelength shifts very similar to FBG2, confirming that FBG2 can reliably serve as a reference sensor. The temperature-induced wavelength shift ($\Delta \lambda_{T}$) measured by FBG2 was used to eliminate the temperature effect from the total wavelength shift of the primary FBG, isolating the strain contribution ($\Delta \lambda_{\epsilon }$):

(9) $$\Delta \lambda_{\epsilon }=\Delta \lambda_{B}-\Delta \lambda_{T}.$$

This compensation method ensured accurate displacement measurements, independent of thermal variations. Despite minor time delays and damping effects probably introduced by the resin, the deviation between the temperature estimated by the FBGs and the reference thermocouple measurements remained within acceptable limits, validating the robustness of this approach.

#### 5.2.3. Displacement Estimation Through FBG Sensors

The estimation of displacement using FBGs involves determining the displacement sensitivity coefficient ($K_{L}$), which relates the strain-induced wavelength shift ($\Delta \lambda_{L}$) to displacement variations (ΔL), as defined by

(10) $$K_{L}=\frac{\Delta \lambda_{B}-\Delta \lambda_{T}}{\Delta L}.$$

Here, $\Delta \lambda_{B}$ is the total wavelength shift measured by the FBG, and $\Delta \lambda_{T}=K_{T}\Delta T$ represents the thermal contribution, subtracted to isolate the strain-induced shift. The change in notation from $K_{\epsilon }$ (strain sensitivity coefficient) to $K_{L}$ (displacement sensitivity coefficient) was made to clearly differentiate between strain and displacement sensitivity: $K_{L}$ connects the wavelength shift ($\Delta \lambda_{L}$) directly to the specimen’s displacement (ΔL). Once $K_{L}$ is determined, displacement variations ($\Delta L_{\mathrm{predicted}}$) are calculated using

(11) $$\Delta L_{\mathrm{predicted}}=\frac{\Delta \lambda_{B}-\Delta \lambda_{T,\mathrm{predicted}}}{K_{L}},$$

where $\Delta \lambda_{T,\mathrm{predicted}}=K_{T}\Delta T_{\mathrm{predicted}}$ is the predicted thermal contribution derived from the reference sensor (FBG2). The accuracy of $\Delta T_{\mathrm{predicted}}$ depends on the calibration of FBG2, ensuring reliable temperature compensation.

**Figure 18:**
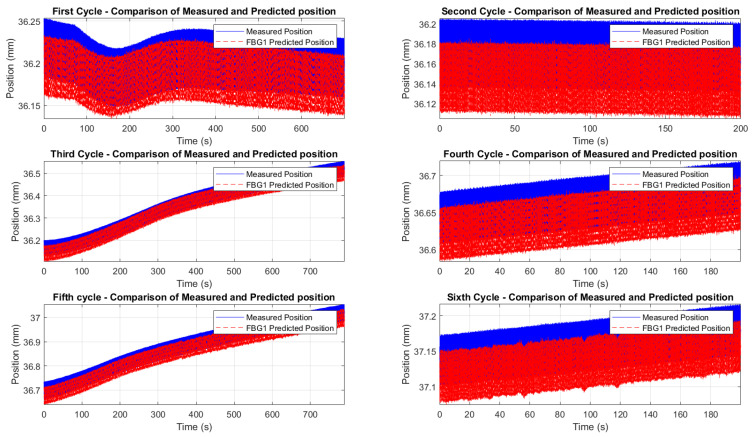
Comparison between measured displacement (blue solid line) and FBG-predicted displacement (red dashed line) during the second thermo-mechanical test. Each subplot represents one of the six consecutive loading–unloading cycles, arranged sequentially from top left to bottom right. The reference sensor (FBG2) was used to compensate for temperature-induced strain shifts, enabling an effective isolation of the displacement signal. Predicted displacements consistently undershoot the Instron crosshead values by a near-constant amount; this downward bias is primarily attributed to a calibration/intercept offset and partial strain transfer through the adhesive layer.

Since no additional stress–strain sensors (e.g., strain gauges) were available on the specimen, comparing the FBG measurements directly to the displacement values measured by the Instron crosshead, rather than stress or strain values, was considered more appropriate. This approach eliminates the risk of introducing additional error through the need to derive stress or strain analytically. The Instron crosshead displacement provided a direct and reliable measurement, ensuring that the FBG-derived displacement values were compared to an accurate reference. The results, shown in Figure 18, demonstrate a good initial agreement between the predicted and measured displacements for all six test cycles. A consistent downward bias between the FBG-predicted displacement and the Instron crosshead (same waveform shape but shifted by a near-constant amount) was observed. We attribute this effect primarily to a calibration/intercept offset and to incomplete strain transfer caused by the resin layer (adhesive shear-lag and partial yielding), combined with the difference between global crosshead motion and the local elongation measured over the FBG gauge length. Residual temperature compensation errors and small timing/synchronization discrepancies may contribute at a lower level. The bias can be corrected in post-processing by subtracting the mean offset or included explicitly as an intercept term in the displacement model.

**Figure 19:**
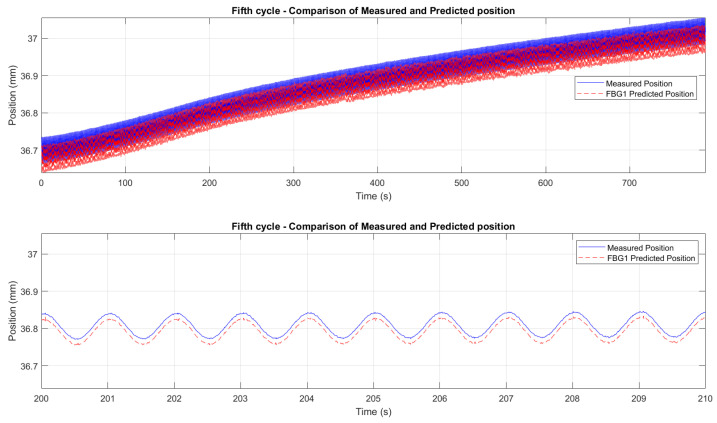
Zoomed comparison between FBG1-predicted displacement (red dashed line) and Instron crosshead displacement (blue solid line) during the fifth cycle (temperature ramp 200–250 °C, cyclic loading 1 Hz). Predicted displacements were obtained after temperature compensation using FBG2 and conversion with the displacement sensitivity coefficients reported in Table 3. The figure highlights excellent waveform agreement while revealing a small, near-constant bias and minor hysteretic behavior attributable to adhesive shear-lag and resin plasticity during peak loads.

**Figure 20:**
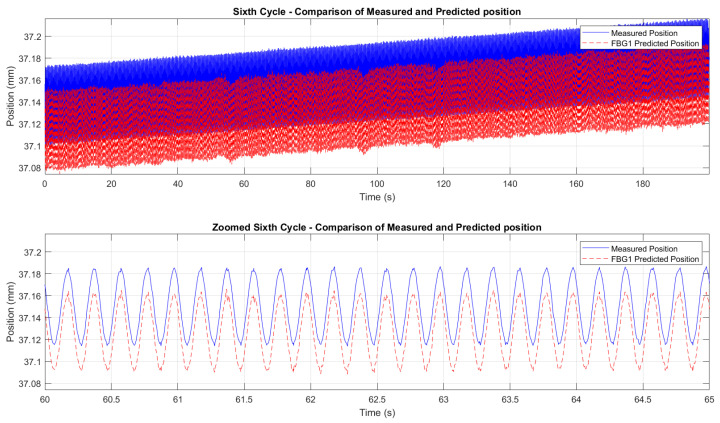
Zoomed comparison between FBG1-predicted displacement (red dashed line) and Instron crosshead displacement (blue solid line) during the sixth cycle (constant 250 °C, cyclic loading 5 Hz). The figure shows preserved amplitude and repeatability after temperature-effect compensation using FBG2, with a slight downward offset of the FBG prediction consistent with incomplete local strain transfer (adhesive compliance/debonding) and small synchronization/compensation residuals. Displacement sensitivity coefficients used for the prediction are reported in Table 3.

To refine the analysis of the wavelength-to-displacement correlation, Figure 19 and Figure 20 provide zoomed comparisons of the predicted displacement values with measured displacements for the fifth and sixth cycles. These detailed views highlight the accuracy of the prediction process, showcasing how the wavelength shifts were accurately converted into displacement measurements. The displacement sensitivity coefficients were determined using displacement readings from the Instron crosshead. The wavelength shift of each FBG was correlated with the displacement measured by the crosshead. This approach eliminates potential biases from the interrogator’s internal calibration, providing a direct sensitivity assessment based on the actual mechanical conditions experienced by the sensors. The determined values of the displacement sensitivity are highlighted in Table 3, for FBG4 and FBG1 during the first test and the second test, respectively. The obtained sensitivity values are consistent with those reported in studies and tests for displacement estimation using FBGs [68,82,83]. Small $K_{L}$ variations across cycles are typical due to factors like misalignments, adhesive behavior, and temperature fluctuations. The resin’s adhesive properties and potential hysteresis may have affected strain transfer, but the overall trends and sensitivity values remain consistent.

The methodology effectively isolates the strain contribution, leveraging the reference sensor (FBG2) to compensate for temperature-induced shifts. These promising outcomes highlight the potential of FBGs as displacement sensors, with room for optimization to achieve even greater accuracy. Results from all of the six cycles also highlighted the importance of selecting a resin capable of withstanding high temperatures to ensure no loss of critical information and to maintain perfect adherence of the optical fiber to the specimen. In our case, when the tension cycles peak at 5 kN, a maximum stress of 80 MPa is generated, exceeding the tensile strength of the resin (54 MPa). This induces a plastic hysteresis behavior of the resin itself, shifting the resin’s behavior from elastic to plastic. This can introduce minor hysteresis in the results, such as the mentioned delay of 30 ms. This effect, though minimal, underscores the need for careful selection of materials that can sustain high mechanical loads without compromising the accuracy of the measurement. A better choice of resin, combined with a better resin application, could lead to even better performance.

## 6. Precision Evaluation

### 6.1. Error Analysis in FBG-Based Temperature Estimation

The precision of the FBG temperature estimation was evaluated by comparing the calibrated FBG readings against reference values measured by a K-type thermocouple throughout the two thermal cycles. This precision was assessed using statistical metrics, specifically the coefficient of determination (R^2^) and the Root Mean Square Error (RMSE). These metrics quantify the goodness of fit and the average prediction error, respectively. Table 4 presents the computed R^2^ and RMSE values for each FBG over the first and second heating cycles. The high R^2^ values (>0.99) and low RMSE values (generally below 5 °C) indicate excellent agreement between FBG-estimated temperatures and the reference thermocouple data, confirming the robustness of the linear calibration and the sensors’ reliability.

RMSE remained below 5 °C across the full 25–300 °C range, corresponding to a maximum relative error of 1.82%, and it could be further reduced with an improved testing setup. These results are encouraging for a preliminary trial, showing strong potential for future applications. With additional tests and refined setups, significant improvements in accuracy and reliability can be achieved.

### 6.2. Error Analysis in FBG-Based Displacement Estimation

This section evaluates the accuracy of FBG sensors in predicting displacement during mechanical loading under varying thermal conditions. Since displacement estimation depends on the differential measurement of Bragg wavelength shifts between sensing and reference gratings—with temperature effects compensated—quantifying the residual error is important to assess the system’s reliability. The RMSE was selected as a simple primary metric, since it provides a direct measure of the mean deviation between the FBG-based predictions and the displacement measurements recorded by the Instron machine. Before evaluating displacement, it was necessary to assess the quality of the temperature compensation. FBG2 was used as the reference sensor for thermal correction. Table 5 compares its temperature estimation to the K-type thermocouple in the first and second mechanical tests. Slightly higher errors were observed compared to the thermal-only tests, likely due to the thermal inertia of the specimen and strain transfer effects through the resin, while the linear fit quality remained high (R^2^ > 0.99).

Cycle-specific errors for FBG2 temperature compensation are detailed in Table 6. RMSE values were computed for each cycle to evaluate FBG2’s performance over time. Cycles with rapid thermal transients exhibited higher RMSE values, highlighting the sensors’ sensitivity to sudden temperature changes, whereas thermally stable cycles showed minimal errors, validating the calibration process under diverse conditions. It is worth noting that the first test exhibited less consistent RMSE values compared to the second test. This discrepancy is likely attributed to the higher thermal inertia of the specimen, which may have continued to transfer heat to the embedded fiber even during thermally stable intervals. Additionally, the thermal setup in the first test may have received less attention in terms of insulation and equilibrium time, resulting in delayed stabilization of the fiber temperature and consequently larger residual errors.

Finally, Table 7 presents the RMSE values for displacement prediction across both tests. Displacement errors remained consistently low (<0.02 mm), even during cycles with rapid thermal transitions or high loading frequencies, confirming the reliability of the FBG sensors.

Following the thermal compensation, the precision of displacement prediction was evaluated by comparing the output of the FBG sensors to the actual position recorded by the Instron actuator. The RMSE values reported in Table 7 quantify the error between the predicted displacement from the FBG sensor signals and the true displacement measured by the Instron system. These errors remained consistently low across all cycles. The low RMSE values (below 0.024 mm in all cases) demonstrate that the sensors maintain accurate displacement tracking even in the presence of thermal fluctuations and rapid mechanical loading.

Despite minor errors introduced probably by the resin or residual temperature effects, the results confirm the robustness of FBG sensors for high-precision displacement monitoring under combined thermal and mechanical conditions. The consistent low RMSE values and effective temperature compensation validate the sensors’ accuracy and reliability in complex environments.

## 7. Conclusions

This study offers a first look at the potential of FBG-based sensors for monitoring key parameters in low-power startup of Generation IV Lead-cooled Fast Reactors. Through early-stage testing and theoretical analysis, the research shows that FBGs are promising candidates for environments with high temperatures and mechanical stress. While further testing, especially in radiative conditions, is needed to fully validate the technology, this work provides a solid starting point for developing durable, integrated fiber optic sensor systems for advanced nuclear reactors.

The analysis of temperature estimation reveals that while the sensors exhibit robust performance, with RMSE values under 5 °C in the 25–300 °C range, further testing in more controlled environments with improved setups and more precise equipment could significantly reduce errors. As seen in the results, most of the observed discrepancies are attributable to specific factors: fan-induced vibrations introduce noise in the FBGs output, the resin’s mechanical and thermal properties limit strain transfer efficiency and contribute to local thermal expansion mismatch, and the thermal inertia of the specimens causes lag in the sensor response relative to the target temperature. With a refined experimental setup, accuracy could be enhanced, making FBGs even more reliable. Displacement monitoring also showed encouraging results, with minimal errors (RMSE around 0.015 mm in the 36.10–37.20 mm range) in cycles with stable thermal conditions. However, the sensors’ performance was slightly affected during rapid thermal changes, likely due to the resin’s influence. These issues could be mitigated with optimized calibration processes, better material choices, and improved sensor integration techniques, ensuring more consistent performance across diverse conditions.

Future research should focus on improving high-temperature resin materials, exploring FBG performance under both radiation and thermal cycling, and carrying out more testing in complex reactor environments. It would be useful to assess long-term operating temperatures and perform extended cycling evaluations, too. These steps are crucial for making real-time monitoring and automated diagnostics a reality. In fact, extrapolation of our results must be made cautiously, as fs-FBG performance in LFR operating environment is not directly validated. The present study establishes a quantitative baseline (sensitivities, RMSE, short-term noise, and bias) under controlled thermo-mechanical conditions, isolating potential failure modes such as adhesive yielding and packaging limits. Overall, these results highlight how fiber optic sensors have the potential to transform reactor monitoring, improving safety, reliability, and efficiency in the process. Focusing on durability and ease of integration for these sensors represents an important step toward enabling future advancements in nuclear reactor monitoring systems.

## Figures and Tables

**Table 1:** Femtosecond-inscribed FBG specifications for the experimental setup.

FBG ID	Wavelength (nm)	Reflectivity (%)
FBG1	1539.64	54.82
FBG2	1544.03	54.45
FBG3	1548.01	54.02
FBG4	1551.94	53.55

**Table 2:** Temperature sensitivity coefficients $K_{T}$ of each FBG sensor for the first and second thermal cycles.

FBG’s ID	First Cycle $K_{T}$ (pm/°C)	Second Cycle $K_{T}$ (pm/°C)
FBG1	12.33	12.37
FBG2	12.43	12.48
FBG3	12.51	12.56
FBG4	12.62	12.61

**Table 3:** Displacement sensitivity coefficients $K_{L}$ of FBG4 and FBG1 sensors during the two thermo-mechanical test cycles.

Cycle	First Test FBG4 $K_{L}$ (nm/mm)	Second Test FBG1 $K_{L}$ (nm/mm)
1	2.03	3.25
2	2.39	3.42
3	2.55	3.58
4	2.64	3.95
5	2.82	2.79
6	2.36	2.37

**Table 4:** Thermal test performance of FBG sensors. The coefficient of determination (R^2^) indicates the quality of the linear fit, while the RMSE quantifies the discrepancies between FBG-estimated temperatures and K-type thermocouple reference values for the first and second thermal cycles.

Sensor	First Cycle R^2^	First Cycle RMSE (°C)	Second Cycle R^2^	Second Cycle RMSE (°C)
FBG1	0.9946	5.20	0.9946	5.06
FBG2	0.9948	5.08	0.9948	4.85
FBG3	0.9959	4.52	0.9959	4.44
FBG4	0.9981	3.12	0.9981	2.98

**Table 5:** Thermal compensation performance of FBG2, showing RMSE against K-type thermocouple measurements and R^2^ of the linear fit for the entire first and second thermo-mechanical tests. FBG2 was not mechanically loaded and served as the temperature reference.

Test	RMSE (°C)	R^2^
First Test	7.28	0.9921
Second Test	4.03	0.9982

**Table 6:** Cycle-specific RMSE values for FBG2 used for temperature compensation, computed for each of the six cycles included in the first and second thermo-mechanical tests.

Cycle	First Test RMSE (°C)	Second Test RMSE (°C)
1	6.35	10.09
2	11.30	5.91
3	9.79	14.29
4	1.40	2.07
5	0.73	5.76
6	1.92	3.98

**Table 7:** Cycle-specific RMSE values of displacement estimation for FBG4 and FBG1, obtained by comparing FBG-predicted positions with the Instron crosshead displacement over the first and second thermo-mechanical tests.

Cycle	First Test FBG4 RMSE (mm)	Second Test FBG1 RMSE (mm)
1	0.0117	0.0164
2	0.0076	0.0239
3	0.0152	0.0141
4	0.0161	0.0219
5	0.0112	0.0180
6	0.0125	0.0234

## Data Availability

The original contributions presented in this study are included in the article. Further inquiries can be directed to the corresponding author.
